# Susceptibility-Related Factor and Biomarkers of Dietary Supplement *Polygonum multiflorum*-Induced Liver Injury in Rats

**DOI:** 10.3389/fphar.2019.00335

**Published:** 2019-04-05

**Authors:** Can Tu, Qin He, Chun-Yu Li, Ming Niu, Zi-Xin Han, Fei-Lin Ge, Yuan-Yuan Zhou, Le Zhang, Xiao-Hui Wang, Jing-Xiao Zhu, Rui-Sheng Li, Hai-Bo Song, Xiao-He Xiao, Jia-Bo Wang

**Affiliations:** ^1^School of Pharmacy, Chengdu University of Traditional Chinese Medicine, Chengdu, China; ^2^China Military Institute of Chinese Medicine, The Fifth Medical Centre, Chinese PLA General Hospital, Beijing, China; ^3^National Cancer Center, National Clinical Research Center for Cancer, Cancer Hospital, Peking Union Medical College, Chinese Academy of Medical Sciences, Beijing, China; ^4^Center for Drug Reevaluation, China National Medical Product Administration, Beijing, China

**Keywords:** idiosyncratic drug-induced liver injury, *Polygonum multiflorum*, susceptibility-related factor, metabolomics, biomarker

## Abstract

*Polygonum multiflorum* [PM, synonym *Reynoutria multiflora* (Thunb.) Moldenke.], a well-known and commonly used Traditional Chinese Medicine and herbal dietary supplement for nourishing the kidney and liver, etc., has aroused wide concern for its reported potential hepatotoxicity. Previous clinical cases and experimental studies have suggested that mild immune stress (MIS) may be one of the susceptibility-related factors of idiosyncratic drug-induced liver injury (IDILI) caused by PM. In this paper, we found that the same dose of PM caused abnormal liver biochemical indicators and liver tissue damage in MIS model rats, while it did not result in liver injury in normal rats, further confirming that MIS is a susceptibility factor for PM-IDILI. Plasma chemokine/cytokine profiling indicated that the MIS model group was significantly different from the other groups, showing a significant upregulation of plasma chemokines, while the MIS/PM group showed upregulated expression of chemokines or pro-inflammatory cytokines. Liver histopathological examination indicated a small amount of inflammatory cytokine infiltration in the MIS group, but no hepatocyte injury, consistent with the plasma profiles of increased chemokines and unchanged inflammatory cytokines. Notably, metabolomics characterization showed that MIS caused reprogramming of these metabolic pathways (such as phenylalanine and glutamate pathways), which was associated with acute phase reactions and inflammatory responses. These results suggested that MIS may promote an immune response to the initial cellular injury induced by PM in the liver, and MIS-induced upregulation of chemokines and metabolic reprogramming may an important mechanism that mediates the susceptibility to PM-IDILI. Furthermore, via receiver operating characteristic (ROC) curves analysis, we identified 12 plasma cytokines (e.g., IP-10, MCP-1 and MIP-1α) and nine metabolomics biomarkers (e.g., L-Phenylalanine, Creatinine, and L-glutamine) with differential capabilities (all ROC AUC > 0.9) of identifying susceptibility model animals from normal ones, which might be of referable value for the clinical recognition of PM-IDILI susceptible individuals.

## Introduction

In recent years, with the widespread use of herbal and dietary supplements (HDS) worldwide, HDS-induced liver injury has become more prominent (Chalasani et al., 2014; Seeff et al., 2015). According to a prospective study by the US Drug-induced Liver Injury Network, HDS are considered the second most common cause of DILI in the United States (Navarro et al., 2014). The LiverTox Database supported by the National Library of Medicine included more than 30 types of herbs that can cause DILI, such as *Polygonum multiflorum* Thunb. (PM) (Teschke et al., 2014). PM is considered to be the most commonly used tonic for liver and kidney. However, reports of liver injury related to PM were significantly increased, which was the most reported liver injury of the single herbs in China (Dong et al., 2014). PM-related IDILI has been reported in more than 30 countries and regions, such as the United States, South Korea, and Japan (Cardenas et al., 2006; Jung et al., 2011; Li et al., 2017). As HDS or traditional medicine, both radix and caulis of PM are widely used to prevent white hair, hair loss, sleep disorder, skin diseases, etc. (Wang et al., 2016). In addition to evidence that PM radix could result in liver injury, reports of liver injury induced by PM caulis have gradually increased in recent years. The clinical cases of PM-DILI have ranged from mild liver injury to severe liver failure, as well as the death of an individual patient. Recently, the National Medical Products Administration has also warned of the risk of liver injury from medicines that contain the caulis of PM. However, the mechanism of PM-related DILI is largely unclear.

Idiosyncratic drug-induced liver injury is a common cause for serious adverse drug reactions in clinical practice (Navarro et al., 2017). Severe IDILI cases may lead to acute liver failure and potentially death (Kaplowitz, 2005; Tujios and Fontana, 2011; Iorga et al., 2017). The occurrence of DILI exhibits significant individual differences, and the susceptibility mechanisms are largely unclear (Roth and Lee, 2017). As DILI cannot be predicted based on dose and pharmacological action, it is difficult to prevent and control the risk of this type of adverse drug reaction in clinical practice (Chen et al., 2015). IDILI is also a major cause of drug development failure, drug withdrawals and post-marketing warnings (Kullak-Ublick et al., 2017; Tujios and Lee, 2018). Therefore, it is of substantial value to screen for predictive biomarkers to recognize susceptible individuals of IDILI and thus subsequently prevent IDILI occurrence by avoiding drug use in susceptible individuals.

Previous clinical case studies and animal experiments have shown that MIS may be one of the susceptibility-related factors of PM-IDILI (He et al., 2017; Meng et al., 2017). In normal rats, PM could not induce obvious liver injury even when administered at a high dose (50 g/kg) for a long time (Tu et al., 2015). In contrast, the double clinical equivalent dose of PM caused significant liver injury in MIS model rats (Li et al., 2015). The phenomenon that MIS promoted the susceptibility of IDILI had also been demonstrated in other drugs and herbs, such as trovafloxacin, psoraleae fructus, and epimedii folium (Waring et al., 2006; Tang et al., 2017; Tian et al., 2017). However, the mechanism of MIS-mediated susceptibility of PM-IDILI remains unclear. In particular, biomarkers for identifying susceptible individuals of PM-IDILI have not previously been found. Therefore, this article employs PM as an example to explore the objectivity of PM-IDILI through an analysis of clinical liver injury cases and experimental evaluation of liver injury on the previously established MIS susceptibility animal model. Furthermore, plasma cytokine/chemokine profiling and metabolomics were used to screen the potential biomarkers associated with the susceptibility factors of PM-IDILI and subsequently attempt to elucidate the potential mechanisms of PM-IDILI susceptibility from the perspective of metabolic reprogramming, linking the disorders of metabolic pathways with the metabolic-immune remodeling in the susceptible state and the subsequent liver injury process. The screened biomarkers would provide a reference for the clinical recognition of PM-IDILI susceptible individuals, as well as understanding the susceptibility factors and mechanism of PM-IDILI.

## Materials and Methods

### Chemicals and Reagents

Methanol and acetonitrile (HPLC grade) were bought from Merck (Darmstadt, Germany). Deionized water was prepared using a Milli-Q water purification system Millipore (Bedford, MA, United States). Other chemicals were all of analytical grade and their purity was above 99.5%. Lipopolysaccharide (LPS, Lot#086M4159V) derived from Escherichia coli 055:B5 and sodium pentobarbital were obtained from Sigma–Aldrich (St. Louis, MO, United States). Alanine transaminase (ALT) and aspartate aminotransferase (AST) analysis kits were purchased from the Jiancheng Bioengineering Institute (Nanjing, China). Cytokine array of Luminex screening and performance assays kit was obtained from R&D Systems (MN, United States). The caulis of *Polygonum Multiflorum* (PM, batch number 10050904) was purchased from Beijing Lv-Ye Pharmaceutical Industries Co., Ltd., and was authenticated by Dr. Xiao-He Xiao (Military Institute of Chinese Materia Medica, Beijing, China). The PM was verified to have met the standards specified by the 2015 edition of Chinese Pharmacopoeia.

*Cis*-2,3,5,4’-tetrahydroxystilbene-2-*O*-β-D-glucoside (*cis*-TSG), emodin-8-*O*-β-D-glucoside and physcion-8-*O*-β-D-glucoside were purchased from Chengdu Chroma-Biotechnology Co., Ltd. (Chengdu, China). *Trans*–2,3,5,4′-tetrahydroxystilbene-2-*O*-β-D-glucoside (*trans*–TSG), emodin and catechin were supplied by Chengdu Pufei De Biotechnology Co., Ltd. (Chengdu, China).

### The UPLC Instrumentation and Conditions

The UPLC was performed by a Waters Acquity Ultra performance liquid chromatograph system equipped with a binary solvent delivery pump (Waters, United States), an auto sample manager, and a photoelectric diode array detector (PDA). Data collection and integration were performed by the Empower 2 software. The chromatographic separation was performed using a Waters Acquity BEH C_18_ column (2.1 mm × 100 mm, 1.7 μm). The mobile phase, consisting of a mixture of acetonitrile (A) and 0.1% (v/v) aqueous phosphoric acid (B), had a flow rate of 0.30 mL min^-1^. The binary gradient elution protocol was as follows: 0∼1 min with 5% A, 1∼9 min with 5–40% A, 9∼19 min with 40–90% A, 19∼21 min with 90–100% A, 21∼23 min with 100% A, 23∼27 min with 100–60% A, 27∼30 min with 60–5% A. The detector wavelength was set at 280 nm. The injection volume was 2 μL and the column temperature was maintained at 30°C.

### Sample Preparation

Dried PM was extracted twice with 10 volumes of 50% ethanol–water (V/V) by cold-soaked extraction for 48 h. After the extraction was completed, the combined extract was filtrated and concentrated under negative pressure at 45°C and freeze-dried to yield a brown ethanol extract. The corresponding concentration was prepared with deionized water prior to use.

### Animals and Experimental Design

Sixty male Sprague-Dawley rats with weights of approximately 180 g were provided by the Laboratory Animal Center of the Academy of Military Medical Sciences (Certification number SCXK-JUN 2007-004). The room temperature was set at 20 ± 2°C, and the humidity was 60–70%. A 12-h day–night cycle was maintained, and the rats had free access to standard diet and water. All animals were acclimated for 3 days prior to the experiments.

The rats were divided at random into six groups (*n* = 10, per group): the normal control rat group (normal); the non-toxic dose of LPS-induced MIS model rat group (MIS); the normal rats treated with low dose (6.75 g/kg, measured as the quantity of the raw herb) PM_1_ group (PM_1_); the normal rats treated with high dose (13.5 g/kg, measured as the raw herb) PM_2_ group (PM_2_); the MIS model rats treated with low dose PM_1_ (MIS/PM_1_); and the MIS model rats treated with high dose PM_2_ (MIS/PM_2_). The rats were injected with LPS (2.8 mg/kg, Sigma) or normal saline in the tail vein by using standard techniques, followed by the intragastric administration of different extracts of PM or an equivalent volume of normal saline 3 h later, then, rats were anesthetized with sodium pentobarbital (50 mg/kg, i.p.) after 7 h later. Blood was collected from the inferior vena cava by the sodium heparin blood collection tube, and the livers were removed from the rats immediately after sacrifice (Li et al., 2015). Food and water were available *ad libitum* for all rats throughout the experiment. The experimental protocol was performed as previously reported (Li et al., 2015; Meng et al., 2017). This study was carried out in accordance with the recommendations of the Institutional Animal Care and Use Committee of 302 Military Hospital. The protocol was approved by the Experimental Animal Welfare and Ethics Center of 302 Military Hospital.

### Plasma Biochemical Analysis

Hepatic injury was estimated by evaluating the activities of ALT and AST in the plasma using assay kits according to the manufacturer’s instructions. The plasma inflammatory state was assessed by cytokine/chemokine profiling (including IL-1α, IL-2, IL-4, IL-5, G-CSF, IL-10, IL-12p70, IL-17A, IL-1β, IL-6, TNF-α, GM-CSF, IL-13, IFN-γ, GRO-α, MCP-1, MIP-1α, Rantes, Eotaxin, MIP-2, IP-10, MCP-3, and VEGF-A), using the Cytokine array of Luminex Assays Kit according to the manufacturer’s instructions.

### Liver Histopathology Assessment

The liver samples were fixed with 10% neutral formalin for 48–72 h, embedded in paraffin after fixation, continuously sectioned at a thickness of 5 μm, stained with hematoxylin and eosin (H&E) and evaluated using a microscope.

The apoptosis of hepatocytes was determined using a TUNEL *in situ* detection kit (Roche, IN, United States) following the manufacturer’s instructions. Five slides from each block were evaluated for the percentage of apoptotic cells using the TUNEL assay. Five watch fields on each section were then randomly selected under a microscope. Positive brown cells and total cells were counted by Image-Pro Plus 6.0 software.

### Plasma Preparation and HPLC-TOF MS Analysis

Prior to the analysis, 300 μl of plasma samples (Normal, MIS, PM_2_ and MIS/PM_2_, groups) were put into individual 1.5 ml tubes separately after being thawed at room temperature. All samples were extracted by adding 900 μl of HPLC grade of acetonitrile, vortex-mixed, centrifuged subsequently for 10 min at 12,000 r min^-1^ at the 4°C. Each supernatant was carefully separate into vials and filtered by 0.22 μm microfiltration membrane for metabolomics analysis. Metabolomics was performed on Agilent Technology iFunnel 6550 Q-TOF LC/MS. Agilent 1200 HPLC system was applied to the liquid chromatogram (LC) analysis. The analysis was conducted with an Agilent ZORBAX SB-C18 column (2.1 × 100 mm, 1.8 μm). The separation was achieved with a 25 min linear gradient with the mobile phases of solvent A (Water spiked with 0.1% formic acid) and solvent B (Acetonitrile spiked with 0.1% formic acid). The flow rate was kept at 0.30 mL/min. The gradient was used as follows: a linear gradient of 100% A over initial–1.0 min, 100–60% A over 1.0–9.0 min, 60–10% A over 9.0–19.0 min, 10–0% A over 19.0–21.0 min, 100% B over 21.0–25.0 min. The temperatures of auto sampler and column were kept at 4 and 30°C, respectively. The eluent was introduced to the mass spectrometer directly.

For mass spectrum (MS) analysis, a high resolution electrospray mass spectrometer (Agilent 6550 Q-TOF/MS) was employed. The electrospray source parameters were fixed as follows: electrospray capillary voltage was 3.0 kV in negative ionization mode and 4.0 kV in positive ionization mode (Li et al., 2016). The mass range was set from m/z 80 to 1000. Gas temperature was 225°C in negative ionization mode and 225°C in positive ionization mode. Gas flow was 13 L/min. Nebulizer was set to 20 pisg (negative) and 20 pisg (positive). Sheath gas temperature was 275°C and sheath gas flow was 12 L/min. Nozzle voltage was 2000 V in both negative and positive mode. For internal mass calibration during the MS analysis, reference masses *m/z* 121.0509 (Purine, C_5_H_4_N_4_) and *m/z* 922.0098 [hexakis-(*1H*,*1H*,*3H*,-tetrafluoropropoxy) phosphazine, HP-0921, C_18_H_18_O_6_N_3_P_3_F_24_] were used in positive mode, and *m/z* 112.9856 [ammonium trifluoroacetate (TFANH4), C_2_O_2_F_3_NH_4_] and *m/z* 1033.9881 (HP-0921, C_18_H_18_O_6_N_3_P_3_F_24_) were used in negative mode. After every five samples a blank sample and a quality control (QC) sample were both analyzed to ensure the stability and repeatability of analysis.

### Biomarker Identification and Metabolic Pathways Analysis

Endogenous metabolites that contributed to the classification were found by variable importance in the projection (VIP) values, which showed the importance of each variable to the classification. Only VIP values > 1 were selected and used for further data analysis. Compounds with significant changes in the groups (*P*-value < 0.05 and fold change > 1.5) were selected as candidate biomarkers. All biomarkers were tentatively identified with the accurate mass-to-charge ratio in Mass Hunter PCDL Manager database and KEGG database ^1^. The pathways analysis of potential biomarkers was performed with MetaboAnalyst 3.0^2^ based on the pathway library of rattus (rat). The ROC analysis was performed with MetaboAnalyst 3.0 based on the biomarker analysis. Independent sample *t*-test was used to reveal the statistical differences of data between two groups. Statistical differences were considered significant when the *P*-value was lower than 0.05.

### Statistical Analysis

The experimental data were expressed as mean ± standard deviation and statistical analysis was performed using SPSS 17.0 (SPSS Inc., Chicago, IL, United States). A value for *P* < 0.05 was considered to be statistically significant. All chromatographic data from plasma was processed by the freely available software package MZmine 2.20^3^, which performed peak noise removal, peak detection, and alignment in an automated and unbiased way using *m/z* data file from Agilent Mass Hunter Work station Data Acquisition. The intensity of each ion was normalized with respect to the total ion count to generate a data matrix, and all these data matrices were introduced to the SIMCA-P 11.0 version (Umetrics AB, Umeå, Sweden) for multivariate statistical analyses including principal component analysis (PCA) and orthogonal partial least-square-discriminant analysis (OPLS-DA) which was utilized to validate the PCA model and identify the differential metabolites. Pathway analysis was performed by the combination of a free web-based tool MetaboAnalyst and the topology with a powerful pathway enrichment analysis.

## Results

### Identification of the Chemical Constituents in PM Extract

In our previous study (Gao et al., 2016), it was reported that the chemical constituents of PM was mainly attributed to the contents of stilbenes and anthraquinones, such as *cis*-TSG, *trans*–TSG, emodin-8-*O*-β-D-glucoside, physcion-8-*O*-β-D-glucoside and emodin. In this paper, a sensitive, precise, and specific Ultra performance liquid chromatography (UPLC) method was established for quantification of six major components in PM (shown in Supplementary Information). According to calculated from calibration curves, the simultaneous determination of major active components including catechin, *cis*-TSG, *trans*–TSG, emodin-8-*O*-β-D-glucoside, physcion-8-*O*-β-D-glucoside and emodin (Supplementary Figure S1), and the relative proportion of compound is 0.10, 0.019, 0.51, 0.071, 0.013, and 0.026%, respectively (Supplementary Table S1).

### Clinical Features of PM Caulis-DILI

**FIGURE 1:**
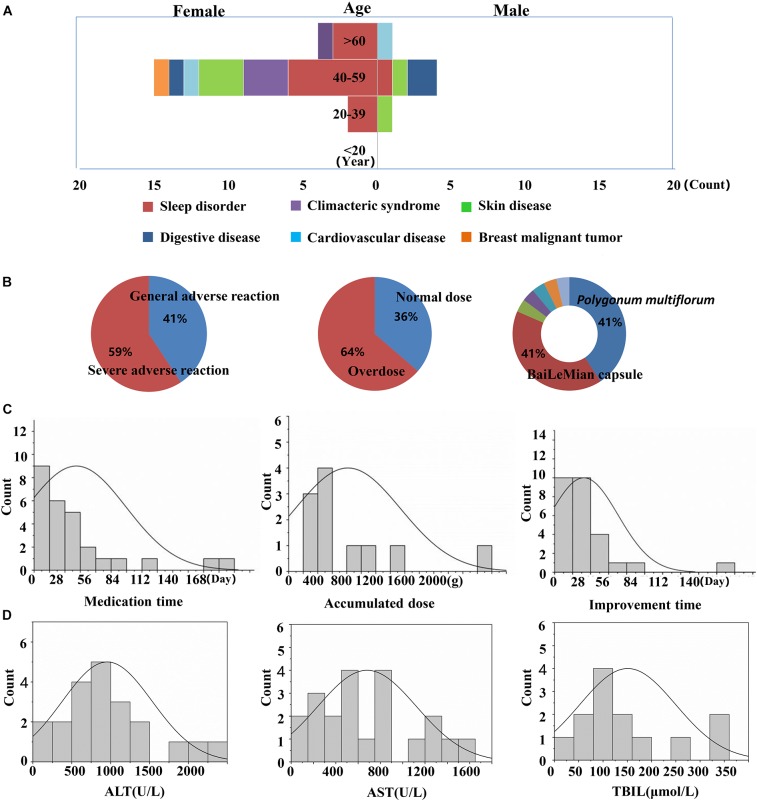
Clinical features of PM-induced liver injury and related risk factors. **(A)** Liver injury cases of gender, age and the purpose of medications; **(B)** degree of adverse drug reaction, dose distribution and drug type distribution; **(C)** time of liver injury for taking the medicine, accumulated dose and improvement time; **(D)** biochemical indicators (ALT, AST, and TBIL).

Twenty-seven cases associated with PM caulis (including its preparations)-DILI were screened out from the National ADR Monitoring database (2012–2016) and were included in this study. The results showed that the ratio of males to females was 1:3.55, the median age of onset was 52 years (range, 18–71 years), and the peak age of DILI occurred at 40 to 59 years of age. The age distribution characteristics of the DILI cases are shown in Figure 1. The age distributions of the DILI cases were as follows: 20–39 years old (11.0%), 40–59 years old (70.0%), and above 60 years old (19.0%). The PM caulis-DILI was more common in women, particularly in the peak age range (40–59 years; *P* < 0.05). The medication purposes of PM caulis included: sleep disorder (45%), skin diseases (14.8%), and climacteric syndrome (11%), etc. The most frequently used preparation was Bailemian capsule (PM caulis as the major component), which accounted for 41% cases overall. Serious adverse reactions occurred in 59% of cases. The median medication time was 16 days, and the median time of improvement after drug withdrawal or treatment was 15 days. Thirty-four percent of cases took the drugs according to the instructions, while 64% of cases were overdose. There was no obvious dose-toxicity relationship between the cumulative dosage and the duration of administration. The clinical biochemical indicators showed that the median values of the ALT, AST and TBIL levels were greater than five times the upper limit of the normal range (ULN). In addition, liver injury often occurred in cases with immune abnormalities, such as sleep disorder, climacteric syndrome and skin diseases, which suggested that the abnormal immune activation of the hosts might be one of the susceptible factors of PM-IDILI.

### MIS Potentiated PM-Induced Liver Injury in Rats

Treatment with PM (PM_1_ or PM_2_) or a non-toxic dose of LPS alone did not cause significant increases in the plasma ALT and AST activities in the rats, compared with the normal group (*P* > 0.05). Co-treatment of the high dose (PM_2_ group) rather than the low dose (PM_1_ group) of PM with a non-toxic dose of LPS resulted in significant increases in both the ALT and AST activities, compared with the MIS group (*P* < 0.01), which indicated that a non-toxic dose of LPS-induced MIS potentiated PM-IDILI in rats.

**FIGURE 2:**
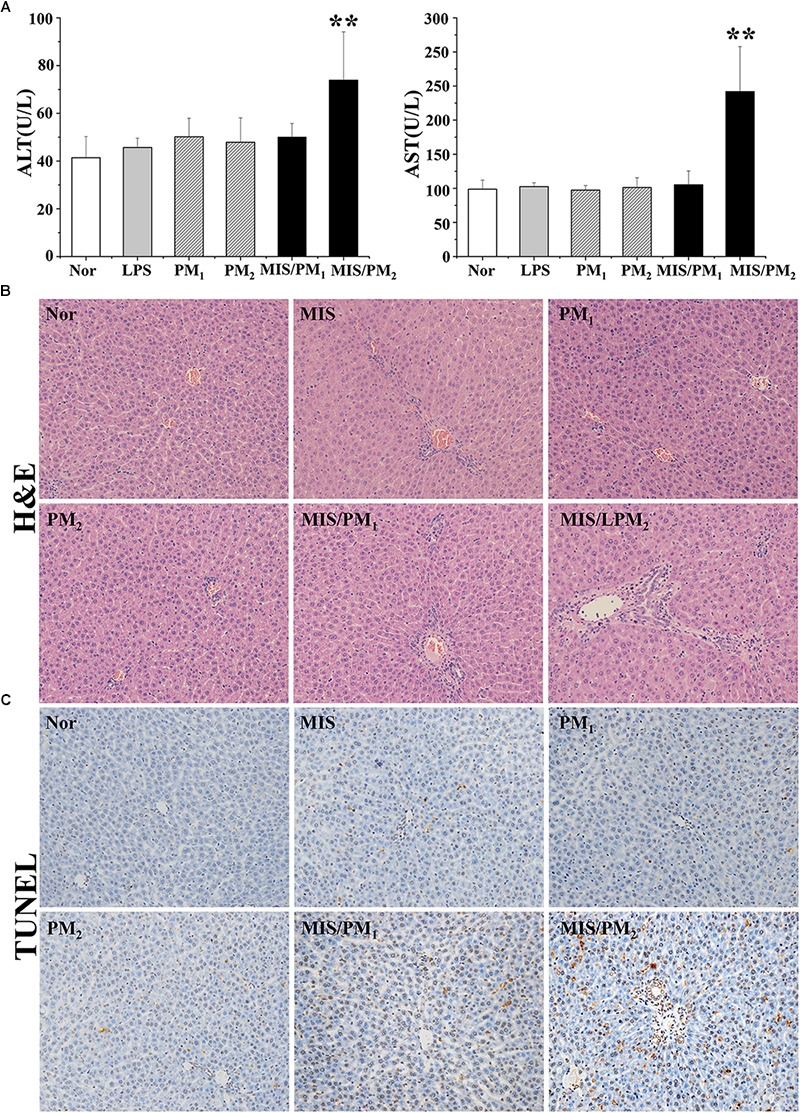
Phenotype of PM-induced liver injury in rats. Rats were randomized into six groups as follows: the normal control rat group (normal); the non-toxic dose of LPS-induced MIS model rat group (MIS); the normal rats treated with low dose (6.75 g/kg, measured as the quantity of the raw herb) PM_1_ group (PM_1_); the normal rats treated with high dose (13.5 g/kg, measured as the raw herb) PM_2_ group (PM_2_); the MIS model rats treated with low dose PM_1_ (MIS/PM_1_); and the MIS model rats treated with high dose PM_2_ (MIS/PM_2_). The results are expressed as the mean ± SD of the rats, and significant differences are indicated (^∗∗^*P* < 0.01 vs. MIS group, *n* = 10). **(A)** The plasma ALT and AST activities. **(B)** Histopathological damage in rat liver (H&E stained, 200× magnification); **(C)** The hepatocyte apoptosis determination by the TUNEL assay.

The H&E staining analysis of the liver sections indicated there were no evident histopathological lesions in the normal or PM (PM_1_ or PM_2_) groups (Figure 2B). In the MIS or MIS/PM_1_ groups, mild inflammatory cell infiltrations were observed in the portal vein area. In the MIS/PM_2_ group, there were significant histopathological changes in the liver sections, including visible swelling, absence of nuclei and focal necrosis of hepatocytes, as well as a mass of inflammatory cell infiltration in the portal vein area around the vessels.

The TUNEL assay showed that there were no or rare apoptotic hepatocytes in the normal or the PM (PM_1_ or PM_2_) groups. In the MIS or MIS/PM_1_ groups, a small number of apoptotic hepatocytes occurred. In the MIS/PM_2_ group, significant and frequent apoptotic hepatocytes were observed, which suggests that high-dose PM induced liver injury in MIS model rats (Figure 2C).

### Correlation Analysis of Cytokines Related to IDILI Susceptibility

It was shown that a non-toxic dose of LPS induced significant increases in the levels of certain chemokines, such as MCP-1, Eotaxin, and IP-10 (*P* < 0.01 or *P* < 0.05, compared to the normal group), rather than obvious hepatocellular injury in the hepatic histopathology assessment. In addition, the plasma chemokine and pro-inflammatory cytokine levels were significantly higher in the MIS/PM groups than in the MIS group. The results suggested that MIS potentiated PM induced liver injury in rats.

**FIGURE 3:**
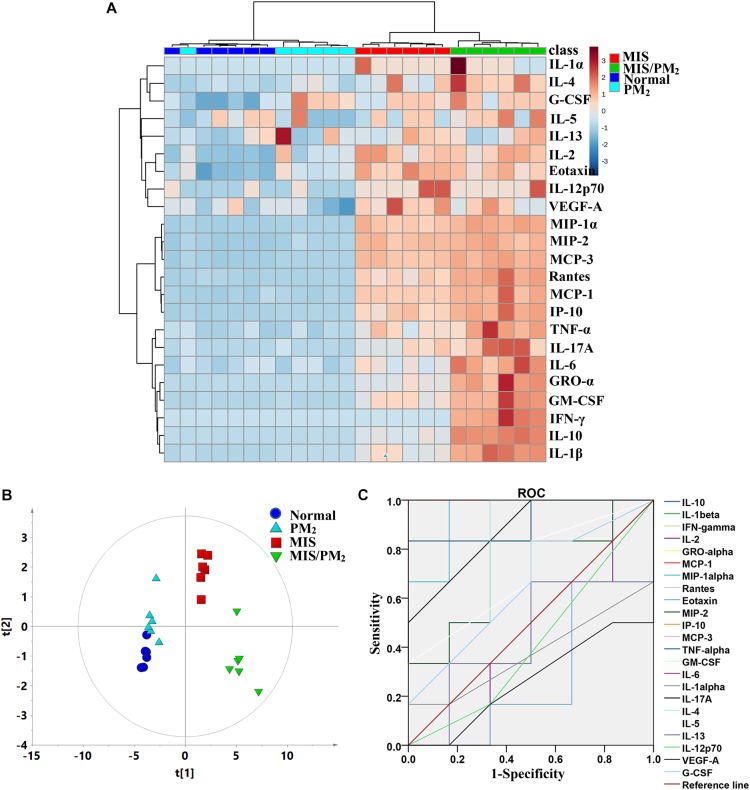
Plasma cytokine expression signatures of PM-IDILI in rats. **(A)** Clustered heat map analysis of plasma cytokines; the colors represent the contents in each of differentially expressed cytokine; **(B)** principal component analysis (PCA) of plasma cytokines; **(C)** receiver operating characteristic (ROC) curves of potential cytokine biomarkers associated with liver injury susceptibility-related factor.

The heat map cluster analysis showed that the four experimental groups had mainly been clustered into two clusters (Figure 3): the first cluster was composed of the MIS group and the MIS/PM group represented by high levels of chemokines and pro-inflammatory cytokines; and the second cluster was composed of the normal group and the PM group represented by low levels of chemokines and cytokines. Furthermore, it could be observed that the chemokines and cytokines had also been divided into two clusters: one cluster was mainly composed of chemokines (such as MCP-1, MIP-α, Rantes, and IP-10), and the other cluster was mainly composed of pro-inflammatory cytokines (e.g., IL-6, IL-1β, and INF-γ). Notably, in the normal group and the PM group, the chemokines or pro-inflammatory cytokines were present at low levels; in the MIS group, the chemokines represented increased levels, while the pro-inflammatory cytokines did not exhibit obvious increases. Moreover, in the MIS/PM group, the chemokines or pro-inflammatory cytokines were present at high levels, and the later indices showed significant increasing trends (compared to the MIS group).

To distinguish the different contributions of various chemokines or cytokines to liver injury, the PCA analyses were performed as an unsupervised statistical method to visualize inter-group inflammatory microenvironment differences. The results showed that the four groups were clearly separated apart, and the susceptibility of PM-induced liver injury could be effectively distinguished by the chemokines and cytokines (Figure 3B). A Receiver Operating Characteristic (ROC) Curve analysis subsequently led to the identification of the changes of cytokines and chemokines (including IL-10, IL-1β, IFN-γ, GRO-α, GM-CSF, IL-6, MCP-3, TNF-α, MCP-1, MIP-1α, Rantes and IP-10) as potential biomarkers associated with PM-IDILI (Supplementary Table S2).

### Metabolomic Profile Analysis of PM-IDILI

**FIGURE 4:**
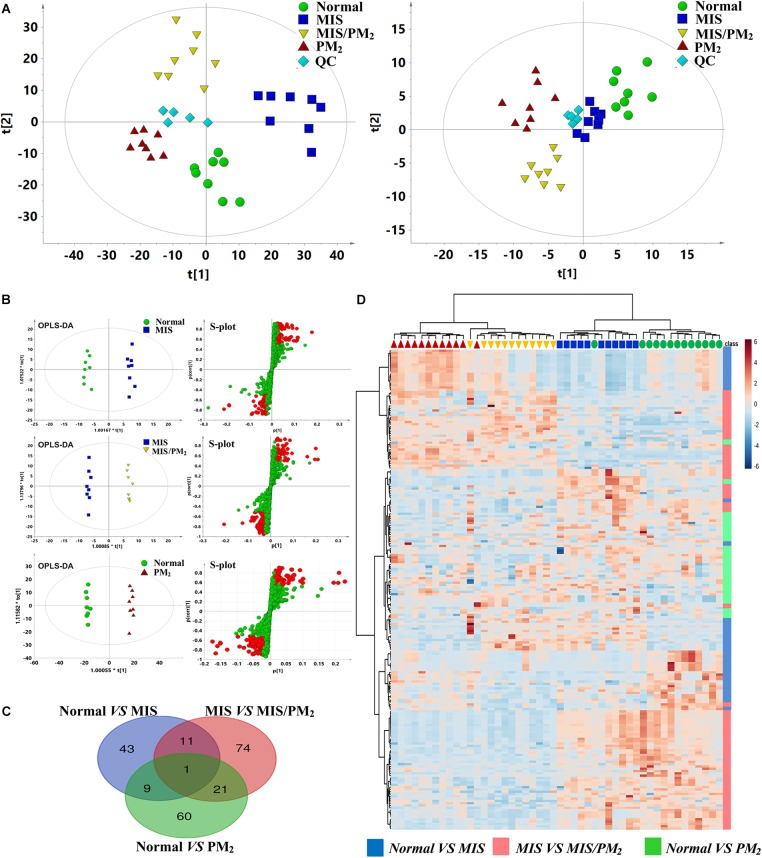
Metabolomic analysis of PM-IDILI in rats. **(A)** PCA score plot of different groups in positive and negative ESI modes. **(B)** OPLS-DA score plot and S-plots displayed for two groups (Normal vs. MIS, Normal vs. PM, and MIS vs. MIS/PM). **(C)** The shared and unique numbers of metabolites were also visualized in Venn diagram from Normal vs. MIS, Normal vs. PM, and MIS vs. MIS/PM. **(D)** Clustered heat map of the 219 significantly changed ions among the normal, MIS, PM, and MIS/PM groups. The colors from blue to red indicate the relative contents of metabolites.

Global metabolic profiles of plasma samples were obtained by HPLC–QTOF/MS in positive and negative ESI modes, and the endogenous metabolites were represented as chromatographic peaks. To better visualize the subtle differences among these complex data sets, multiple pattern recognition methods were applied to differentiate the phenotype from the plasma metabolome of the rats. An unsupervised PCA statistical method was employed to assess the metabolic differences. The score plots of the PCA analysis derived from the ESI– mode and ESI+ mode data are shown in Figure 4, respectively. The QC samples clustered closely in both ESI mode score plots, which suggests the stability of the LC/MS system throughout the whole analysis. An obvious separation trend was observed among the normal, MIS, PM and MIS/PM groups in both PCA models, which indicates a considerable difference in the metabolome among these groups. Moreover, the MIS/PM group was far away from the rest of the groups, which suggests the changes in the metabolic profile that resulted from PM-induced liver injury may be significantly different from other groups.

To further screen the potential candidate biomarkers associated with the susceptibility of PM induced DILI, the supervised statistical method of OPLS-DA and S-plot were applied to better understand the different metabolic patterns and resulted in the identification of potential biomarkers that were significantly changed in these groups. The biomarkers related to the susceptibility factors of PM-IDILI were further screened. Based on the established OPLS-DA and S-plot models, the normal vs. PM, normal vs. MIS, and MIS vs. MIS/PM were compared and analyzed separately. There were 219 ions with significant differences (VIP > 1 or |*p*(corr)|≥ 0.5). Analysis by Venn diagrams showed that there were 43 (normal vs. MIS), 74 (MIS vs. MIS/PM) and 60 (normal vs. PM) differential ions within these independent comparisons. From the heat map cluster analysis, according to the Pearson correlation coefficient clustering, the changes of different colors of metabolites represented the difference of the content changes among the different groups, suggesting that these differential ions could be used to effectively distinguish the four groups. In the next step, the previously screened differential metabolites were identified by the accurate mass-to-charge ratio in the Mass Hunter PCDL Manager online database. The true Mass tolerance mass error was limited to 10 ppm, and mass spectrometry was subsequently performed. The structure of the identified metabolites was analyzed and verified. The *m/z*, retention time and structural formula of the eligible differential metabolites are listed in Supplementary Table S3.

### Correlation Network and Metabolic Pathway of Differential Metabolites

**FIGURE 5:**
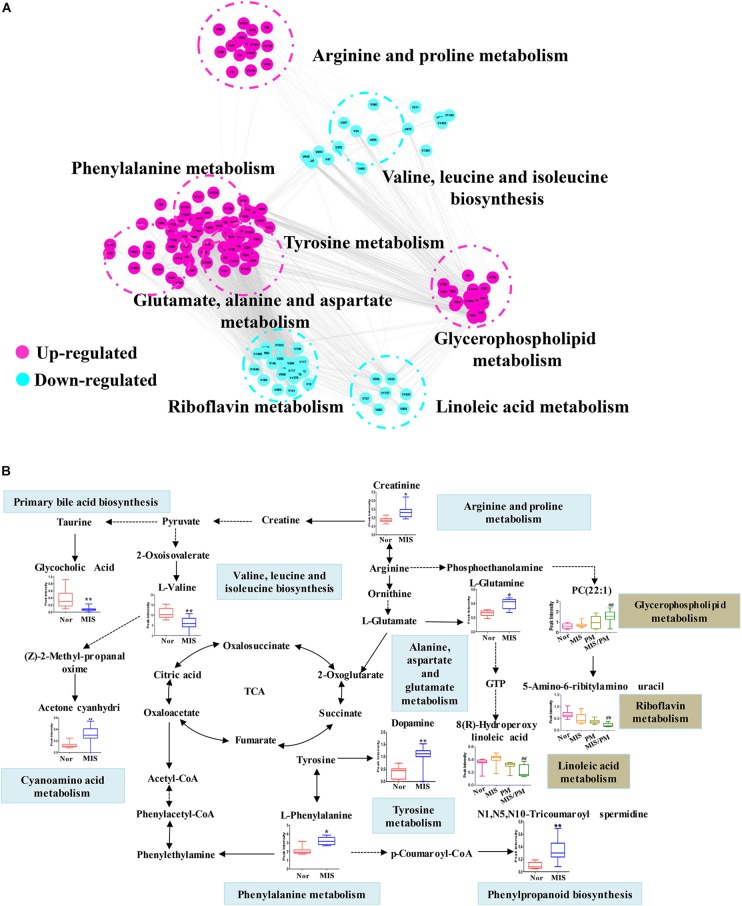
Metabolic correlation networks and metabolic pathway of differential metabolites. **(A)** Highly correlated metabolites (PCC > 0.6) are connected with a line. Red and green represent upregulated and downregulated metabolites, respectively. **(B)** Metabolic network of the significantly changed metabolites. The normalized contents are shown under the chemical name. Blue and red bar charts indicate normalized content in the normal and LPS groups, respectively. Significant differences are indicated (^∗^ *P* < 0.05, ^∗∗^ *P* < 0.01 vs. normal group; ^##^ *P* < 0.01 vs. MIS group).

To investigate the potential relationship between different metabolites, Cytoscape software was used to establish a metabolic network of PM-induced liver injury and its susceptibility factors. To obtain a global metabolomic view of PM-induced liver injury, all differential metabolites (219 ions) were included in the network. As shown in Figure 5A, the mainly altered metabolic pathways lay in amino acid metabolism, including the upregulation of phenylalanine, tyrosine and glutamine metabolic pathways and the downregulation of valine metabolic pathways, which might be highly correlated with the susceptibility of PM-induced liver injury. In addition, significant changes in metabolic pathways, such as riboflavin, linoleic acid, and phosphoric acid, were observed after PM administration on the basis of the MIS model, which suggests a high correlation with PM-induced liver injury. Based on the knowledge of these different metabolites and the online database of metabolic pathways, the map of the metabolic pathways related to the susceptibility of PM-induced liver injury was further established (Figure 5B). The data indicated that MIS mediated the remodeling of several metabolic pathways. For example, phenylalanine metabolites (L-phenylalanine), tyrosine metabolites (dopamine), arginine metabolites (creatinine), and glutamate, alanine and aspartate metabolites (L-glutamate) were upregulated. Valine, leucine and isoleucine metabolites (L-valine) and primary acid synthesis metabolites (glycocholic acid) were downregulated. The results suggested that the remodeling of these metabolic pathways reflects the effect of IDILI susceptibility factors in biological networks. In addition, changes in the metabolic pathways in the MIS/PM vs. MIS group were observed, such as significant downregulation of riboflavin metabolites (5-amino-6-ribitylamino uracil), as well as linoleic acid metabolites [8(*R*)-hydroperoxylinoleic acid] and upregulation of glycerophospholipids metabolites (PC (22:1(13Z)/14:0), which suggests that the drug could induce changes in the phenotype of liver injury based on the susceptibility factors.

### Screening for Susceptibility-Related Biomarkers of PM-Induced Liver Injury

**FIGURE 6:**
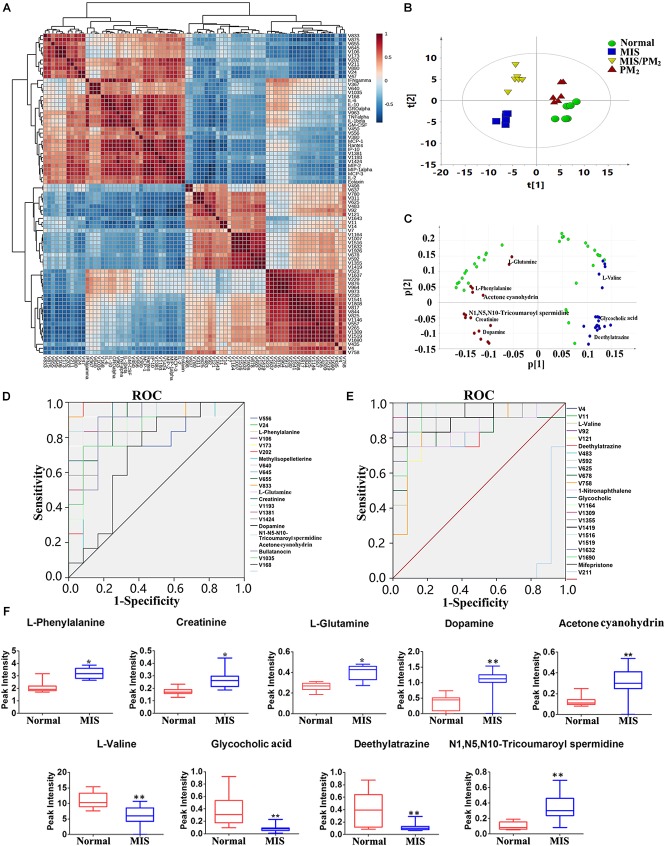
Screening for susceptibility-related biomarkers of PM-IDILI. **(A)** Pearson correlation coefficient of the differential metabolites and cytokines. **(B)** PCAof differential metabolites. **(C)** Loading scatter plot of differential metabolites; red and blue represent positive and negative, respectively. **(D,E)** ROC curves of potential biomarkers associated with liver injury susceptibility-related factors. **(F)** Content changes of high value identified biomarkers, including L-phenylalanine, Creatinine, L-glutamine, N1, N5, N10-tricoumaroyl spermidine, Dopamine, Acetone cyanohydrin, L-valine, glycocholic acid and deethylatrazine (^∗^*P* < 0.05, ^∗∗^*P* < 0.01 vs. normal group).

The Pearson correlation coefficient heat map was constructed using the 43 differential metabolites and chemokines/cytokines screened out in the previous analyses. The differential metabolites were selected (the correlation coefficient PCC > 0.6 with susceptible cytokines) as candidate biomarkers highly correlated with the susceptibility of liver injury (Figure 6A,B). The results showed that there were two types of metabolites highly correlated with the previously screened chemokines and cytokines (Figure 6C). The metabolites with positive correlations (red dot) and negative correlations (blue rot) were significantly separated. Predictive performance analysis and the ROC curves were performed (Figure 6D,E). The ROC AUC values, sensitivity and specificity are shown in Supplementary Table S4. Nine metabolites had an AUC value > 0.9, including L-phenylalanine, creatinine, L-glutamine, N1, N5, N10-tricoumaroyl spermidine, dopamine, acetone cyanohydrin, L-valine, glycocholic acid and deethylatrazine. The content changes of these nine potential biomarkers are shown in Figure 6F. Compared with the normal group, the contents of six biomarkers were significantly increased, while the content changes of the other three biomarkers were decreased. In addition, unidentified differential metabolites with a high predicted performance are shown in Supplementary Table S4. Although these metabolites highly correlated with the susceptibility of IDILI were not identified, they provide a basis for future investigations on the potential application value of the clinical identification of susceptible individuals with liver injury.

### Screening for Liver Injury Related Biomarkers of PM-IDILI

A heat map analysis of 74 differential metabolites associated with liver injury and the chemokines/cytokines related to susceptibility was performed as shown in Supplementary Figure S2A: The candidate biomarkers related to liver injury were further screened, and a Pearson’s correlation coefficient diagram was adopted. The differential metabolites related to the susceptibility factor (the correlation coefficient PCC > 0.6) were selected as the highly correlated metabolites. The ROC curve was used to test the predictive performance of the selected biomarkers for liver injury (Supplementary Figure S2B and Supplementary Table S5). Totally three metabolites were screened out as potential biomarkers (AUC values > 0.9) as follows: 5-amino-6-ribitylamino uracil, 8(*R*)-hydroperoxylinoleic acid and QH2. Compared with the MIS group, the contents of three metabolites were significantly decreased in the MIS/PM group (Supplementary Figure S2C).

## Discussion

With the widespread usage of HDS, accompanied by reports of the potential hepatotoxicity of HDS has increased tremendously worldwide, not only in Asian countries (such as China, Japanese, Korea, etc.), but also in the western countries (Navarro et al., 2017). Individuals who consume these HDS usually consider to the natural or be unaware of the potential side effect. Besides, it is not generally known among the public that natural medicines, such as Dictamni Cortex, Psoraleae Fructus, and Epimedii Folium have been associated with DILI (Teschke et al., 2016; Jing and Teschke, 2018). However, HDS-induced liver injury is often unpredictable based on dose and pharmacological action, and affects only susceptible individuals (Chen et al., 2015). Therefore, it is a challenging and frontier issue in toxicological studies to elucidate the susceptibility mechanisms of IDILI and screen potential biomarkers of susceptible individuals.

In this study, the clinical case analysis showed that the occurrence of liver injury caused by PM caulis existed only in very few patients and was not dose- or time-dependent (Figure 1), which represented typical IDILI characteristics. The clinical characteristics are similar to those of PM radix induced liver injury. In addition, we reproduced the results of previous studies on PM-IDILI in animal models, that is, there was no significant liver injury phenotype solely via PM administration; in contrast, PM led to an obvious liver injury phenotype on the basis of a non-toxic dose LPS-induced MIS rat model, represented by significant increases in liver functional biochemical indicators (ALT and AST, *P* < 0.01), high expression of pro-inflammatory cytokines, and substantial inflammatory cell infiltration and liver cell apoptosis in liver tissues (Figure 2). Interestingly, only upregulation of chemokines, rather than pro-inflammatory cytokines, was observed in non-toxic LPS-induced MIS model rats (Figure 3A), accompanied by mild infiltration of immune cells in the liver portal area and little apoptotic hepatocytes (Figure 2B,C). Thus, the results suggested that the upregulation of cytokines may increase individual sensitivity to hepatotoxic drugs by expanding the inflammatory responses of the immune system to drug initial lesions.

There are difficulties in predicting the occurrence of IDILI by serum enzyme (e.g., serum ALT, AST, ALP or other enzymes), although these markers are extremely useful, but are of limited sensitivity and specificity, they were only applicable once injury was substantial and established (Laverty et al., 2010; Chen et al., 2015). Therefore, the identification of novel biomarkers that are both sensitive and specific to the liver would have great benefit. The current theory suggests that the majority of idiosyncratic drug reaction have evidence to support immune involvement. Changes in levels of circulating cytokines and chemokines have been proposed as possible biomarkers of DILI (Lacour et al., 2005). Indeed, several cytokines or small groups of cytokines (e.g., IL-1β, TNF-α, and IL-6) have been reported to be altered in a few experimental studies of DILI (Steuerwald et al., 2013). Previous study showed that levels of IL-10 and IL-4 gene polymorphisms can promote Th2-mediated antibody responses to increase the susceptibility to diclofenac liver injury (Aithal et al., 2004). In this study, the results revealed that MIS could significant increase the levels of certain chemokines and pro-inflammatory cytokines, such as MCP-1, IP-10, IL-1β, and TNF-α. The upregulation of cytokines may increase individual sensitivity to hepatotoxic drugs by expanding the inflammatory responses of the immune system to drug initial lesions (Takayama et al., 2011). IDILI-associated drugs sensitize hepatocytes to cell death signaling from cytokines, which is processed and released through NF-κB pathway or NLRP3 inflammasome activation (Mangan et al., 2018), and stimulate macrophages, neutrophils, or other cell types to release considerably numerous cellular factors and inflammatory mediators, generating a series of inflammatory reactions and liver injury (Roth and Ganey, 2011). It has also been reported that high expression levels of cytokines associated with innate immunity may be associated with a poor prognosis of DILI (Laverty et al., 2010; Steuerwald et al., 2013). The chemokines and cytokines (such as MCP-1, MIP-1α, IL-1β, and TNF-α) or their combinations were therefore used to identify candidate biomarkers for PM-IDILI susceptible individual recognition.

Metabolism and immunity are interdependent and highly integrated systems, and their balance is the key to maintaining the host’s normal state (Buck et al., 2017). The researchers have been showed that the function of immune cells was a functional product determined by its metabolic state (Murray et al., 2015). Recent studies have revealed that the metabolic state of activated immune cells was distinct from non-activated immune cells, and different types of immune cells had divergent characteristics of metabolic pathway activation or inhibition (Jha et al., 2015). In this study, the results demonstrated that the metabolic profiles had significantly changed (Figure 5), but the pathological phenotype changes were not observed in the MIS group (Figure 2). Thus, from the perspective of metabolomics, the expression levels of endogenous metabolites were more sensitive than phenotypic changes to characterize the effects of the susceptibility factors of IDILI. Furthermore, the upregulation of the phenylalanine, tyrosine, arginine and proline, and glutamate, alanine and aspartate metabolism pathways and downregulation of the valine, leucine and isoleucine metabolism pathways (*P* < 0.01, compared with the normal group) were the main metabolomics features in the susceptible model animal group (Figure 5A). Glutamine metabolism has been found to play a role in the function of several immune cells (Fu et al., 2018), including macrophages and T lymphocytes. Previous study also confirmed that arginine metabolism was altered in association with acute hepatic injury in rats, and plasma arginine was possibly specific biomarkers for liver injury (Saitoh et al., 2014). Therefore, the mechanism of MIS-mediated susceptibility of PM-IDILI can be understood from the perspective of metabolic reprogramming based on metabolomics.

Amino acid metabolic redistribution is used to synthesize proteins involved in inflammation and immune responses (Floc’h et al., 2004), as well as important compounds involved in immune cell proliferation and other immune responses (Nakaya et al., 2014). The acute phase proteins are rich in aromatic amino acids (such as phenylalanine), and the inflammatory response increases the concentrations of acute phase proteins in the plasma, while the amino acid synthesis ratios of the corresponding proteins significantly increase (Reeds et al., 1994; Rittig et al., 2016). In this experiment, the results revealed that the plasma levels of L-phenylalanine and L-glutamine in the MIS group were significantly increased, which may be related to the massive synthesis of acute phase proteins in the state of inflammatory stress. Evidence has suggested that glutamine is essential for antigen presentation, phagocytosis and the synthesis of many cytokines (such as TNF-α, IL-1β, and IL-6) in macrophages and other types of immune cells. Glutamine-dependent tricarboxylic-acid cycle is the principal source of succinate, as a metabolite in innate immune signaling, which enhances IL-β production during inflammation (Tannahill et al., 2013). Taken together, the metabolic reprogramming of the host caused by the susceptibility factors of MIS may make the host’s immune function tend to develop toward the susceptible state (high expression of chemokines), although it did not cause an obvious inflammatory reaction and liver injury phenotype, which suggested the synergistic effect of susceptibility factors and drugs on liver injury.

Furthermore, on the basis of MIS modeled susceptibility factors, the PM caulis led to a significant liver injury phenotype, accompanied by high expression of pro-inflammatory cytokines in the plasma, and inflammatory immune cell infiltration and an inflammatory response were observed in liver tissues (Figure 2). Metabolite identification and pathway enrichment analysis indicated that the metabolic profiles of the liver injury induced by PM were mainly shown as follows: downregulation of the riboflavin (vitamin B2) and linoleic acid pathway, upregulation of the glycerophospholipid pathways (Figure 6). In this study, a significant change in 5-amino-6-ribitylamino uracil might be associated with the immune inflammatory response, as it was reported that 5-amino-6-ribitylamino uracil could selectively activate mucosa-associated constant T cells, the secretion of various cytokines, and directly or indirectly participate in the host’s immune response (Corbett et al., 2014). In addition, the content of linoleic acid was significantly reduced in the MIS model group (*P* < 0.01, compared to the normal group), which suggests that the consumption of linoleic acid might be involved to produce pro-inflammatory mediators in the immune activation progress. It has been reported that when the host was stimulated by mild inflammation, linoleic acid would be released from phospholipids, leading to the formation of inflammatory mediators and mediation of the inflammatory response (Santoro et al., 2013; Ma et al., 2016).

## Conclusion

**FIGURE 7:**
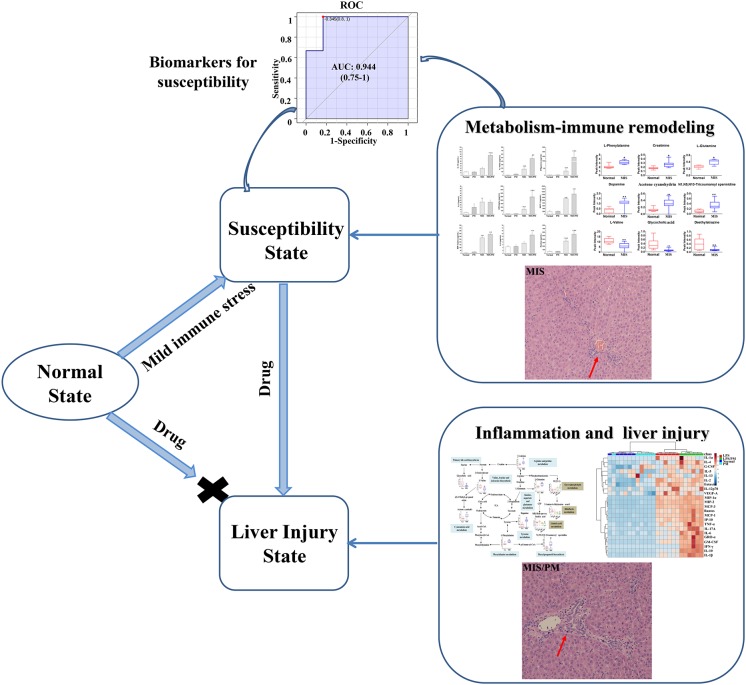
Mechanism of susceptibility-related factors and liver injury related to *Polygonum multiflorum* based on metabolism-immune remodeling.

In summary, this study confirmed the idiosyncratic characteristic of PM-induced liver injury from clinical cases and MIS animal models. Besides, the susceptibility mechanisms of liver injury induced by PM may regulate immune inflammation and metabolic dysfunctions (Figure 7). based on immune inflammation and the metabolic disorder pathways associated with susceptibility factors, we screened 12 chemokines related to inflammation and immune regulation (such as IP-10, MCP-1, and MIP-1α) and nine metabolites related to inflammation and immune regulation (such as L-phenylalanine, L-glutamine and creatinine) as potential biomarkers to identify susceptible model animals from the normal ones (ROC AUC > 0.9). Although there is not a clinical verification, this research also provided a new insight for the safety study of herbal and traditional medicines which might be all belong to idiosyncratic toxicity.

## Author Contributions

CT, QH, and C-YL performed the experiments, analyzed the data, and wrote the manuscript. MN, Z-XH, and F-LG collected and prepared the samples. Y-YZ, ZL, X-HW, and J-XZ performed the analyses. R-SL and H-BS amended the manuscript. X-HX and J-BW designed the study and amended the manuscript.

## Conflict of Interest Statement

The authors declare that the research was conducted in the absence of any commercial or financial relationships that could be construed as a potential conflict of interest.

## Footnotes

1 www.genome.jp/kegg/

2 http://www.metaboanalyst.ca/

3 http://mzmine.sourceforge.net/

## Supplementary Material

Figure S1 Chemical structures and chromatograms (UPLC) of *Polygonum Multiflorum* extract. **(A)** Chemical structures of six target compounds (I catechin,II *cis*-2,3,5,4′-tetrahydroxystilbene-2-*O*-β-D-glucoside (*cis*-TSG), III *trans*–2,3,5,4′-tetrahydroxystilbene-2-*O*-β-D-glucoside (*trans*–TSG), IV emodin-8-*O*-β-D-glucoside, V physcion-8-*O*-β-D-glucoside, and VI emodin). **(B)** Chromatograms of six mixed reference standards. **(C)** Chromatograms of sample of PM from Hubei (the relative proportion of catechin, *cis*-TSG, *trans*–TSG, emodin-8-*O*-β-D-glucoside, physcion-8-*O*-β-D-glucoside and emodin is 0.10, 0.019, 0.51, 0.071, 0.013, and 0.026%, respectively).

Figure S2 Screening for potential liver injury metabolites biomarkers of PM-IDILI. **(A)** Pearson correlation coefficient of the differential metabolites and cytokines. **(B,C)** The receiver operating characteristic (ROC) curves of potential biomarkers associated with liver injury of PM. **(D)** The content changes of high value identified biomarkers, including 8(*R*)-hydroperoxylinoleic acid, QH2 and 5-amino-6-ribitylamino uracil (^∗∗^ *P* < 0.01 vs. MIS group).

TABLE S1 Calibration curves for six standard compounds.

TABLE S2 The ROC curve of potential cytokines biomarkers associated with PM-IDILI.

TABLE S3 Identification and trends of change of differential metabolites.

TABLE S4 The ROC curve of susceptibility-related metabolites biomarkers for PM-IDILI (AUC > 0.9).

TABLE S5 The ROC curve of liver injury metabolites biomarkers for PM-IDILI (AUC > 0.9).
