# Supplementary figures and images for: Susceptibility-Related Factor and Biomarkers of Dietary Supplement Polygonum multiflorum-Induced Liver Injury in Rats
Source: Front Pharmacol. 2019 Apr 5;10:335. doi: 10.3389/fphar.2019.00335 (PMC6459954; doi:10.3389/fphar.2019.00335)

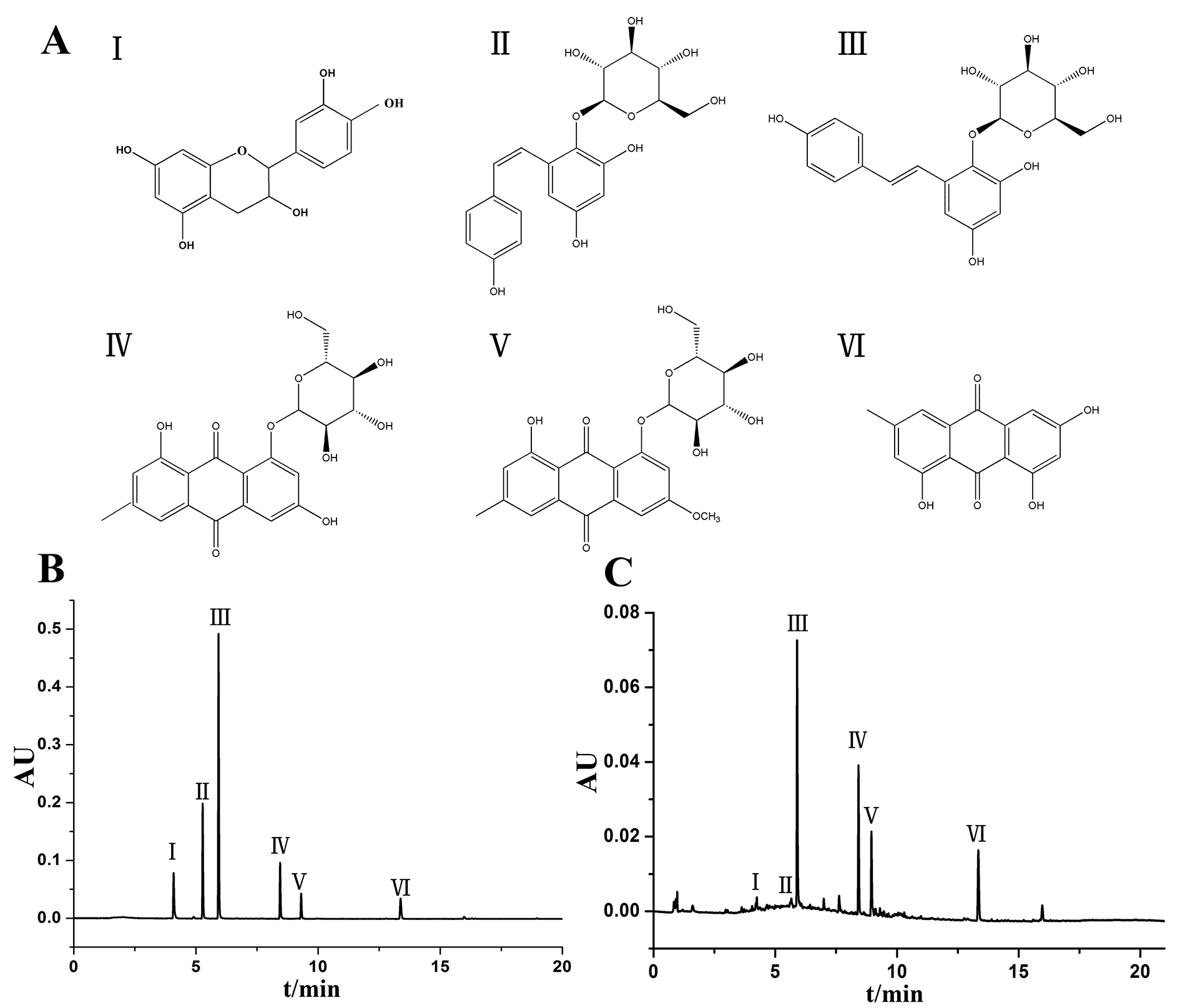

Supplement: Figure S1 — Chemical structures and chromatograms (UPLC) of Polygonum Multiflorum extract. (A) Chemical structures of six target compounds (I catechin,II cis-2,3,5,4′-tetrahydroxystilbene-2-O-β-D-glucoside (cis-TSG), III trans–2,3,5,4′-tetrahydroxystilbene-2-O-β-D-glucoside (trans–TSG), IV emodin-8-O-β-D-glucoside, V physcion-8-O-β-D-glucoside, and VI emodin). (B) Chromatograms of six mixed reference standards. (C) Chromatograms of sample of PM from Hubei (the relative proportion of catechin, cis-TSG, trans–TSG, emodin-8-O-β-D-glucoside, physcion-8-O-β-D-glucoside and emodin is 0.10, 0.019, 0.51, 0.071, 0.013, and 0.026%, respectively). [file Image_1.TIF]

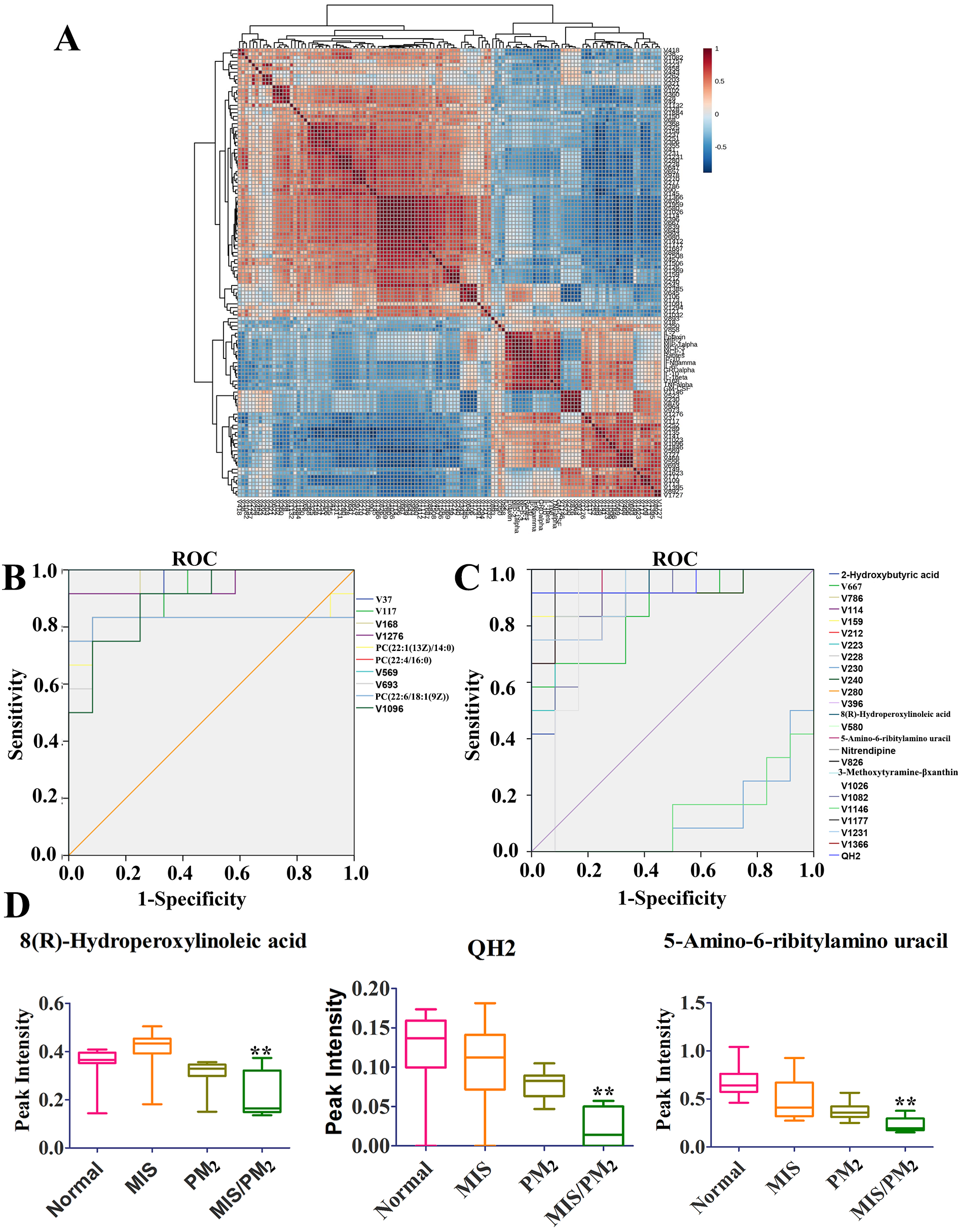

Supplement: Figure S2 — Screening for potential liver injury metabolites biomarkers of PM-IDILI. (A) Pearson correlation coefficient of the differential metabolites and cytokines. (B,C) The receiver operating characteristic (ROC) curves of potential biomarkers associated with liver injury of PM. (D) The content changes of high value identified biomarkers, including 8(R)-hydroperoxylinoleic acid, QH2 and 5-amino-6-ribitylamino uracil (∗∗ P < 0.01 vs. MIS group). [file Image_2.TIF]
