# Supplementary material for: Susceptibility-Related Factor and Biomarkers of Dietary Supplement Polygonum multiflorum-Induced Liver Injury in Rats
Source: Front Pharmacol. 2019 Apr 5;10:335. doi: 10.3389/fphar.2019.00335 (PMC6459954; doi:10.3389/fphar.2019.00335)
Supplement: TABLE S1 — Calibration curves for six standard compounds. [file Presentation_1.pdf]

***Supplementary materials for*：**

Susceptibility-related Factor and Biomarkers of Dietary Supplement *Polygonum Multiflorum*-Induced Liver Injury in Rats

Can Tu[1, 2‡], Qin He[2‡], Chun-Yu Li[3], Ming Niu[2], Zi-Xin Han[2], Fei-Lin Ge[2], Yuan-Yuan Zhou[2], Le Zhang[2], Xiao-Hui Wang[2], Jing-Xiao Zhu[2], Rui-Sheng Li[2], Hai-Bo Song[4], Xiao-He Xiao[2*] and Jia-Bo Wang[2*]

1 School of Pharmacy, Chengdu University of Traditional Chinese Medicine, Chengdu, 610000, China;

2 China Military Institute of Chinese Medicine, the Fifth Medical Centre, Chinese PLA General Hospital, Beijing,100039, China;

3 National Cancer Center/National Clinical Research Center for Cancer/Cancer Hospital, Chinese Academy of Medical Sciences and Peking Union Medical College, Beijing, 00021, China;

4. Center for Drug Reevaluation, NMPA, Beijing, 100022, China;

Supplemental the Phytochemical Analysis Procedures

1. Preparation of sample solutions

The PM samples were pulverized and sifted through an 80 mesh screen. 2g of powder was then extracted by ultrasonication with 30 mL of 50% (v/v) ethanol for 1 h. After being cooled at the room temperature, the sample was compensated with 50% ethanol for any loss during this process. The solution was filtered through a 0.22μm filter membrane before being injected to HPLC analysis. All sample solutions were stored at 4℃.

2. Preparation of standard solutions

Stock solutions of individual standard (catechin, *cis*-TSG, *trans*-TSG,) at a certain concentration were prepared by dissolving the compounds in methanol and stored at 4℃. Other standards (emodin-8-*O*-β-D-glucoside, physcion-8-*O*-β-D-glucoside and emodin) were dissolved in DMSO, then being dissolved in methanol at a certain concentration and stored at 4℃.Working solution of all the standards mixture was compounded immediately before analyses by diluting the stock solutions with the required concentrations. The solutions of standards were all prepared in dark brown volumetric flasks.

Validation of the UPLC method

Results

1 Precision

To confirm the precision of the UPLC method. The chromatographic peak area of each component to be measured was recorded by taking the same mixed standard solution for 6 times continuously at 1.0 μL each time. The results showed that the RSD of the chromatographic peak area of 6 standards solution (such as catechin, *cis*-TSG, *trans*-TSG, emodin-8-*O*-β-D-glucoside, physcion-8-*O*-β-D-glucoside and emodin) are 1.69%, 0.75%, 1.28%, 1.40%, 1.70% and 1.37%, respectively.

2. Stability

Stability was determined with one sample solution that was analyzed at 0 h, 2 h, 4 h, 8 h, 10 h, 12 h, and 24 h at room temperature. The results showed that the RSD of the chromatographic peak area of 6 standards solution (such as catechin, *cis*-TSG, *trans*-TSG, emodin-8-*O*-β-D-glucoside, physcion-8-*O*-β-D-glucoside and emodin) are 1.36%, 1.43%, 1.03%, 2.67%, 1.96% and 1.29%, respectively.

3. Repeatability

To confirm the repeatability of the method, six independently prepared solutions from the same sample were tested. Samples were prepared using the method outlined in section "Preparation of Sample Solutions". The repeatability was analyzed by the RSDs of relative peak areas of six compounds were 0.69%, 0.61%, 0.56%, 2.45%, 2.29% and 1.81%, respectively.

4 Calibration curves

The standards of calibration curves were plotted with a series of concentrations of above mixed standard solutions. Each concentration was analyzed in triplicate for an average value and calibration curves were constructed in the form of the regression equation y = ax + b, where y and x are the peak area and the concentration of the compounds. The calibration curves were shown in Table S1.

Table S1 Calibration curves for six standard compounds

Referance substance	Rt/min	Regression equation	r	Linear range/ (μg·mL^{-1})
Catchin	3.902	Y=6891.2X-16353	0.9999	32.59~195.56
Cis-TSG	5.116	Y=10754X-352.81	0.9992	8.33~83.33
Trans-TSG	5.759	Y=13571X+363786	0.9995	130.67~784.00
Emodin-8-*O*-β-D-glucoside	8.267	Y=29071X+14887	0.9999	16.23~97.40

Physcion-8-*O*-β-D-glucoside	9.131	Y=28082X+12282.2	0.9999	6.93~41.60
Emodin	13.157	Y=35998X-6400.6	0.9997	11.33~68.00

5. Recovery

The Recovery test was carried out by adding a known amount of mixed reference standard solutions into a certain amount of PM .The mixture was extracted as described in “Preparation of Sample Solutions”, and analyzed using the established UPLC method. The recovery value was calculated according to the following formula: recovery (%) = (amount determined - amount original)/amount spiked ×100%. The recovery was analyzed by the RSDs of relative content of six compounds (such as catechin, *cis*-TSG, *trans*-TSG, emodin-8-*O*-β-D-glucoside, physcion-8-*O*-β-D-glucoside and emodin) were 2.19%, 1.86%, 1.86%, 1.40%, 0.93% and 1.33% ,respectively.

Table S2 The ROC curve of potential cytokines biomarkers associated with PM-IDILI (AUC>0.9).

**Biomarkers**	**AUC**	**Sensitivity**	**1-Specificity**	**Sens.+ （1-Spec.）**
IL-10	1	1	1	2
IL-1β	1	1	1	2
IFN-γ	1	1	1	2
GRO-α	1	1	1	2
MCP-1	1	1	1	2
Rantes	1	1	1	2
IP-10	1	1	1	2
GM-CSF	1	1	1	2
IL-6	1	1	1	2
MIP-1α	0.94444	1	0.833333	1.83333
MCP-3	0.94444	0.833333	1	1.83333
TNF-α	0.91667	0.833333	1	1.83333

Table S3 Identification and trends of change of differential metabolites

No.	TR (min)	m/z	Formula	Metabolites	log2(FC)	Pathway
V47	1.85	165.0790	C9H11NO2	L-Phenylalanine	0.65186	Phenylalanine metabolism
V211	1.35	153.0790	C8H11NO2	Dopamine	1.5139	Tyrosine metabolism
V450	1.46	85.0528	C4H7NO	Acetone cyanohydrin	1.2739	Cyanoamino acid metabolism
V880	1.13	113.0589	C4H7N3O	Creatinine	0.64126	Arginine and proline metabolism
V380	19.8	583.2682	C34H37N3O6	N1,N5,N10-Tricoumaroyl spermidine	1.7736	Phenylpropanoid biosynthesis
V875	1.12	146.0691	C5H10N2O3	L-Glutamine	0.60154	Alanine, aspartate and glutamate metabolism
V367	7.58	155.131	C9H17NO	Methylisopelletierine	0.82804	Tropane, piperidine and pyridine alkaloid biosynthesis
V468	17.55	788.6169	C44H87NO8P	PC(22:1(13Z)/14:0)	1.0558	Glycerophospholipid metabolism
V556	17.54	810.6013	C46H85NO8P	PC(22:4(7Z,10Z,13Z,16Z)/16:0)	1.4148	Glycerophospholipid metabolism
V727	18.45	832.5856	C48H83NO8P	PC(22:6(4Z,7Z,10Z,13Z,16Z,19Z)/18:1(9Z))	1.2366	Glycerophospholipid metabolism
V1007	9.63	465.309	C26H43NO6	Glycocholic acid	-2.2629	Primary bile acid biosynthesis
V311	7.58	187.0625	C6H10ClN5	Deethylatrazine	-1.7702	Atrazine degradation
V14	1.07	117.079	C5H11NO2	L-Valine	-0.86459	Valine, leucine and isoleucine biosynthesis
V780	7.05	173.0477	C10H7NO2	1-Nitronaphthalene	-1.7508	Metabolism of xenobiotics by cytochrome P450
V203	1.09	104.0481	C4H8O3	2-Hydroxybutyric acid	-0.97433	Propanoate metabolism
V667	5.55	360.1375	C18H20N2O6	3-Methoxytyramine-β-xanthin	-0.87383	Betalain biosynthesis
V647	9.45	276.1177	C9H16N4O6	5-Amino-6-ribitylamino uracil	-1.1309	Riboflavin metabolism
V457	13.56	312.2287	C18H32O4	8(*R*)-Hydroperoxylinoleic acid	-0.87694	Linoleic acid metabolism

V1369	17.73	320.1915	C19H28O4	QH2	-1.9106	Oxidative phosphorylation

Table S4 The ROC curve of susceptibility-related metabolites biomarkers for PM-IDILI (AUC>0.9)

No.	Biomarkers（m/z）	AUC	sensitivity	specificity	Sens.+（1-Spec.）
**Positive**					
V47	L-Phenylalanine	0.9583	1.0000	0.9167	1.9167
V880	Creatinine	0.9583	0.9167	0.9167	1.8333
V875	L-Glutamine	0.9514	0.9167	0.9167	1.8333
V380	N1,N5,N10-Tricoumaroyl spermidine	0.9514	0.9167	0.9167	1.8333
V833	241.0921	0.9444	0.9167	1.0000	1.9167
V24	119.0738	0.9375	1.0000	0.9167	1.9167
V202	102.0472	0.9375	1.0000	0.9167	1.9167
V106	329.3945	0.9306	0.9167	0.8333	1.7500
V173	658.8898	0.9306	0.9167	0.8333	1.7500
V211	Dopamine	0.9306	0.9167	0.9167	1.8333
V645	658.8399	0.9236	0.9167	0.8333	1.7500
V450	Acetone cyanohydrin	0.9028	0.9167	0.9167	1.8333
**Negative**					
V1309	543.5511	0.9861	1.0000	0.9167	1.9167
V4	543.3346	0.9722	1.0000	0.8333	1.8333
V1007	Glycocholic acid	0.9722	0.9167	1.0000	1.9167
V1164	411.2459	0.9722	0.9167	0.9167	1.8333
V92	234.1581	0.9653	0.9167	0.9167	1.8333
V1519	506.2390	0.9653	0.8333	1.0000	1.8333
V483	220.1796	0.9514	0.8333	0.9167	1.7500
V1516	411.2696	0.9514	0.9167	0.9167	1.8333
V1690	1087.0730	0.9514	0.8333	1.0000	1.8333
V1926	429.2668	0.9514	0.9167	0.9167	1.8333
V1632	487.2533	0.9444	0.9167	0.9167	1.8333
V121	117.0795	0.9306	0.8333	0.8333	1.6667
V311	Deethylatrazine	0.9097	0.7500	1.0000	1.7500
V11	L-Valine	0.9097	0.7500	1.0000	1.7500
V14	463.2869	0.9097	0.9167	0.8333	1.7500
V1419	543.3346	0.9861	1.0000	0.9167	1.9167

Table S5 The ROC curve of liver injury metabolites biomarkers for PM-IDILI (AUC>0.9)

**No.**	**Biomarkers(*m/z*)**	**AUC**	**sensitivity**	**specificity**	**Sens.+（1-Spec.）**
**Positive**					
V569	230.0776	1.0000	1.0000	1.0000	2.0000
V1276	302.0461	0.9514	0.9167	1.0000	1.9167
V37	158.1322	0.9375	0.8333	0.9167	1.7500
V117	202.1208	0.9306	0.8333	0.9167	1.7500
V168	120.9554	0.9167	1.0000	0.7500	1.7500
**Negative**					
V114	329.2583	1.0000	1.0000	1.0000	2.0000
V396	329.2550	1.0000	1.0000	1.0000	2.0000
V580	448.1859	1.0000	1.0000	1.0000	2.0000
V667	360.1375	1.0000	1.0000	1.0000	2.0000
V826	492.2113	1.0000	1.0000	1.0000	2.0000
V839	360.1348	1.0000	1.0000	1.0000	2.0000
V1026	448.1851	1.0000	1.0000	1.0000	2.0000
V1366	580.2218	1.0000	1.0000	1.0000	2.0000
V1959	492.2108	1.0000	1.0000	1.0000	2.0000
V1177	580.2617	0.9722	0.9167	1.0000	1.9167
V457	8(*R*)-Hydroperoxylinoleic acid	0.9653	1.0000	0.9167	1.9167
V1369	QH2	0.9514	1.0000	0.9167	1.9167
V228	276.1333	0.9444	0.9167	0.8333	1.7500
V786	1227.3184	0.9306	0.9167	0.8333	1.7500
V280	276.1138	0.9306	0.7500	1.0000	1.7500
V647	5-Amino-6-ribitylamino uracil	0.9306	0.7500	1.0000	1.7500
V1231	276.1173	0.9236	1.0000	0.7500	1.7500
V223	148.0115	0.9167	0.9167	0.8333	1.7500
V159	285.2222	0.9097	1.0000	0.8333	1.8333
